# A theoretical study of the global and local electrophilicity, nucleophilicity, polarizability and QTAIM theory for calix[4]arene-gas interaction

**DOI:** 10.1016/j.heliyon.2020.e04554

**Published:** 2020-07-31

**Authors:** B. Gassoumi, H. Ghalla, R. Ben. Chaabane

**Affiliations:** a Laboratory of Advanced Materials and Interfaces (LIMA), University of Monastir, Faculty of Science of Monastir, Avenue of Environnment, 5000, Monastir, Tunisia; b University of Monastir, Quantum and Statistical Physics Laboratory, Faculty of Science, Monastir, 5079, Tunisia; c Institute of Light and Matter, UMR5306 University of Lyon 1-CNRS, University of Lyon, 69622, Villeurbanne Cedex, France

**Keywords:** Physical chemistry, Theoretical chemistry, Calix[4]arene, Hydrogen bond, Electro-philic and nucleophilic sites, Polarizability, Interaction energies

## Abstract

The calix[4]arene molecule, abbreviated as CX[4], is known by the four phenolic groups and a hydrophobic cavity able to enclose small molecules. The interactions between CX[4] and NO_3,_ NO_2_, CO_2_, and N_2_ gas molecules have been studied. These guest species are placed inside and outside the cavity of the host molecule CX[4]. The formation of H-bonding has been deeply discussed based on the infrared spectrum and the polarizability analysis. Global and local indices have been calculated for a series of gas (NO_3,_ NO_2_, CO_2_ and N_2_) in interaction with the CX[4] molecule to explain the electrophilic or nucleophilic activations in endo-vs. exo-cavity interaction zone. As expected, there is a correlation between the proposed global electrophilicity and global nucleophilicity together for an explanation of the chemo-selectivity region. Finally, the topological parameter analyses of the host-guests interactions have been estimated by using DFT calculations.

## 1 Introduction

The selectivity of the anionic or cationic guests in microscopic or macroscopic systems facilitates the recognition of the magnetic and electrostatic properties of the several guests’ complexes [1, 2, 3, 4, 5]. The CX[4] exhibit a hydrophobic cavity form [6] and a specific chemical composition. Moreover, the CX[4] is characterized by a specific height and a diameter, these two parameters facilitate the complexation with small molecules [7, 8, 9]. The specific cavity of the CX[4] has attracted the experimenters and may be used in the medical [10, 11, 12] or micro-biological field [13, 14]. In the literature, there are several works which discuss the interaction of CX[4] with small molecule (CH_4_) and gas molecules (NH_3_ and C_2_H_2_) [5, 15, 16, 17]. Herein, we have studied the physical and chemical properties of the CX[4]-NO_3_, CX[4]-NO_2_, CX[4]-N_2_ and CX[4]-CO_2_ complexes (The specific gas in the endo or exo-cavity position). We have discussed the interactions between the CX[4] molecule and NO_3_, NO_2_, CO_2_ and N_2_ gases outside or inside the cavity. The encapsulation of these gases may be a good subject for pollution. The NO_3_, NO_2_, CO_2_ and N_2_ gases have been chosen in our study because they can form a dipole-dipole or CH…π hydrogen-bonding interactions with CX[4].

By using DFT calculations, we have described the dynamic stabilities of the endo-vs. exo-cavity of the CX[4]-gas complexes. The nucleophilicities and electophilicities distribution sites of these host-guests complexes have been performed. The vibrational properties of the CX[4]-gas complexes have been studied. The polarizability study of the stable host-guests has explained the transfer of the charge between the gases to the π-electron of the phenol ring. The recognition of the weak or the strong and the nature of the interactions of such guest with a cage molecule have been analyzed by the AIM topological parameters.

## 2 Computational details

The optimization of CX[4] and CX[4]-gas have been performed by the Density Functional Theory (DFT) method by using the global hybrid generalized gradient approximation B3LYP [15, 18, 19, 20] coupled to the D3BJ (empirical Becke and Johnson damping dispersion corrections) in combination with the 6-31+G(d) basis set, as implemented in a Gaussian 09 package [21] and the GaussView [22] as a visual program. The binding energies have been calculated taking into account the Basis Set Superposition Error (BSSE) counterpoise correction (CP) according to the formalism of Boys and Bernardi [23].

The binding energies (E_b_) are given by the following formula:

(1) $$\Delta E_{CX\left[4\right]-gas}=E_{CX\left[4\right]-gas}-E_{CX\left[4\right]}-E_{gas}+BSSE$$

where E_CX[4]-gas_, E_CX[4]_ and E_gas_ are the total energies of the host-guest and host or guest molecules. The reactivity parameters based on the Fukui function have been calculated in the same framework. The infrared spectrum and the polarizability of these studied compounds have been analyzed by the DFT/B3LYP-D3BJ method. The different topological parameters have been calculated using the Bader's atoms in molecules (AIM) theory by the AIM2000 program [24,25].

## 3 Results and discussions

**Figure 1:**
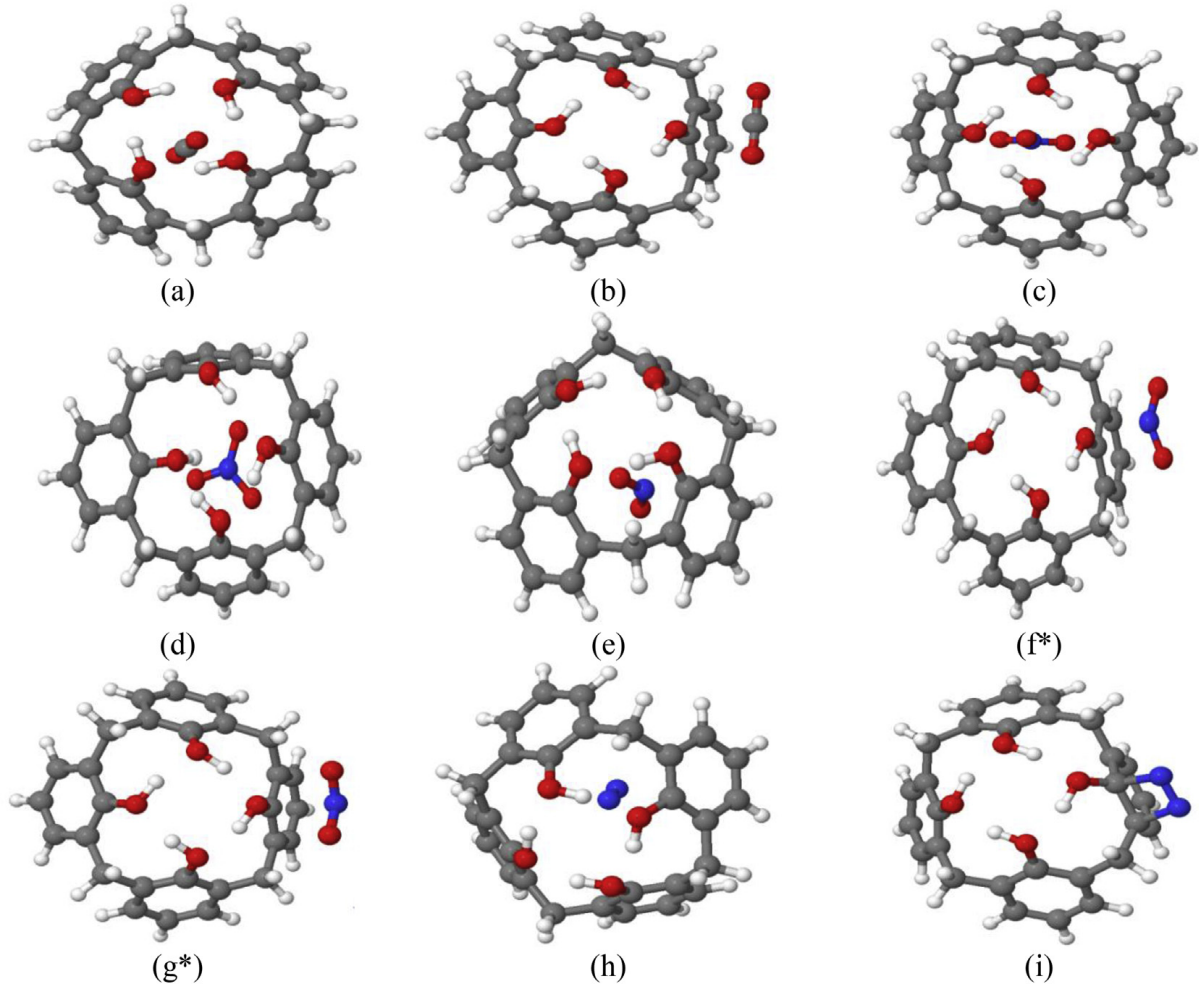
Optimized geometries of the CX[4]-gas (CX[4]-CO_2(endo)_ (a), CX[4]-CO_2(exo)_ (b), CX[4]-NO_3(paral.)_ (c), CX[4]-NO_3(perp_._)_ (d)_,_ CX[4]-NO_2(endo)_ (e), CX[4]-NO_2(exo)_ (f∗), CX[4]-NO_2(exo)_ (g∗), CX[4]-N_2(endo)_ (h) and CX[4]-N_2(exo)_ (i)) structures using B3LYP-D3BJ/6-31+G(d) method (Top view).

**Figure 2:**
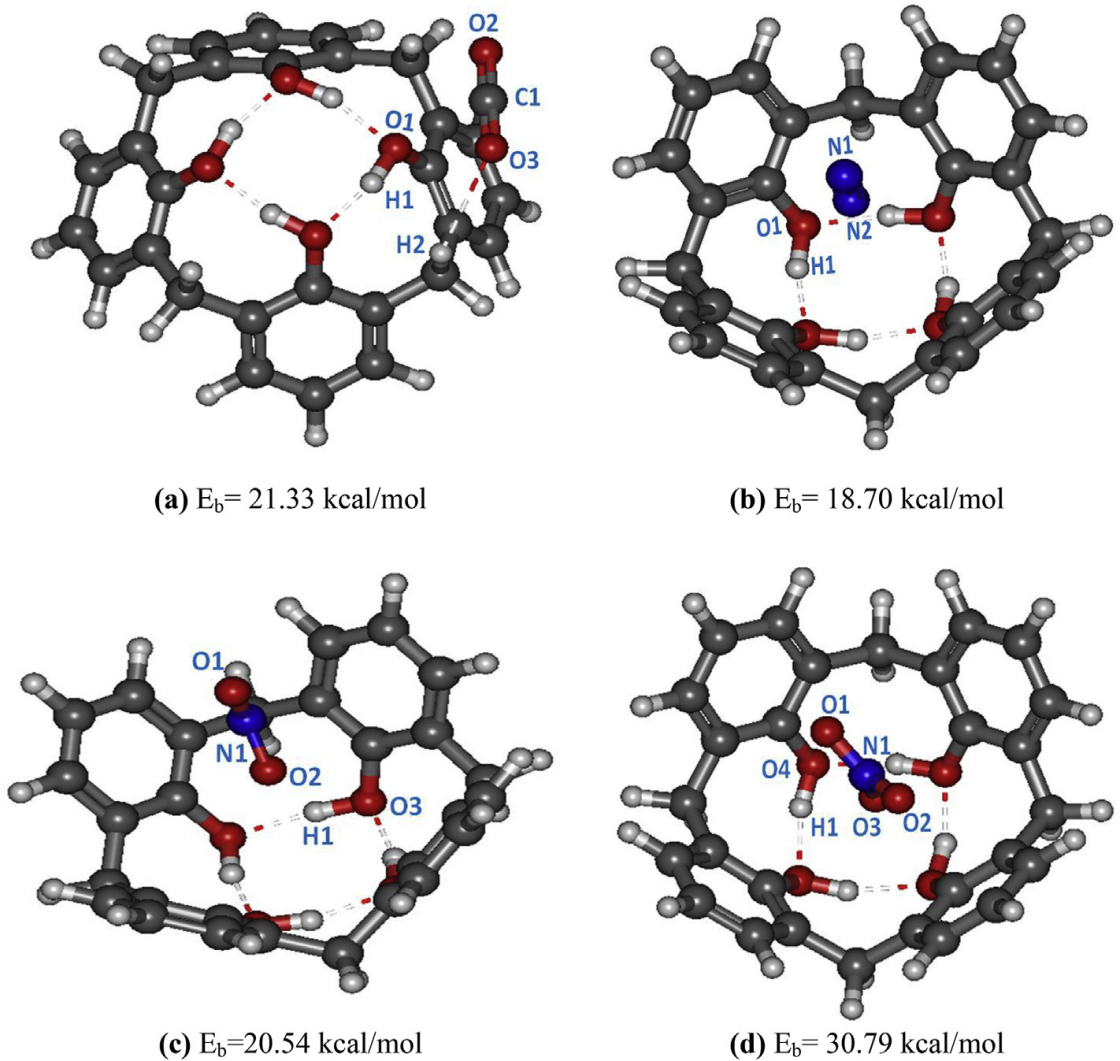
Optimized geometries of the stable CX[4]-gas (CX[4]-CO_2_ (a), CX[4]-N_2_ (b), CX[4]-NO_2_ (c)_,_ and CX[4]-NO_3_ (d)) structures using B3LYP-D3BJ/6-31+G(d) method (Top view).

The CX [4]-gas complexes have been optimized at B3LYP-D3BJ/6-31+G(d) level of theory, and shown in Figures 1 and 2. The NO_3_, NO_2_, CO_2_ and N_2_ gases have been studied in the endo and exo-cavity positions. The highest binding energy value (21.33 kcal/mol) has been obtained for CX[4]-CO_2_ complex.

The binding energy values are listed in Table 1. Obviously, the CX[4]-CO_2(endo)_ complex is more stable than CX[4]-CO_2(exo)_. Based on the dipole-dipole interactions between the CX[4] and the CO_2_ gas, the CX[4]-CO_2(endo)_ complex has the larger stability. The NO_3_ gas may be placed in the exo or endo-cavity positions. In this context, we have shown that the interaction of the NO_3_ gas outside the cavity is very weak (There is a divergence).

**Table 1:** Binding energy (in (kcal/mol)) of CX[4]-gas complexes without E_b_ and with BSSE correction.

Complexes	E_b_	BSSE	E_b_ (with BSSE)
CX[4]-NO_3__(//)_	24.62	6.17	30.79
CX[4]-NO_3(+)_	16.51	6.05	22.56
CX[4]-N_2(exo)_	16.89	1.70	18.59
CX[4]-N_2(endo)_	16.90	1.80	18.70
CX[4]-NO_2(exo)f∗_	18.17	1.82	19.99
CX[4]-NO_2(exo)g∗_	18.11	1.80	19.91
CX[4]-NO_2(endo)_	17.83	2.71	20.54
CX[4]-CO_2(endo)_	18.62	2.71	21.33
CX[4]-CO_2(exo)_	18.10	2.71	20.81

Moreover, we have calculated tow complexes of the CX[4]-NO_3_, NO_3_ perpendicular to the 4-fold axis of CX[4] and the NO_3_ gas parallel to the 4-fold axis. The CX[4]-NO_3(+)_ is characterized by a very weak energy in comparison with the CX[4]-NO_3(//)_. The E_b_ energy of the CX[4]-NO_3(//)_ is equal to 22.56 kcal/mol. The CX[4]-NO_3(//)_ have a larger binding energy than CX[4]-N_2(exo/endo),_ CX[4]-CO_2(exo/endo)_ and CX[4]-NO_2(exo/endo)._ In the CX[4]-NO_3(+)_ complex, we have noted a C4 symmetry. In addition, the formation of the low dipole moment between the CX[4] and the NO_3(//)_ gas imposed a larger stability for this host-guest. In this context, the orientation of the O⋯H groups does not change during the interaction of the CX[4] with the NO_3_ gas. For the CX[4]-N_2_ complex, we have optimized two geometries based on the position of the N_2_ in order to show the stable host-guest. First, the N_2_ gas is located outside of the cavity. Second, this gas located perpendicular to the 4-fold axis of CX[4]. From Table 1, we conclude that the binding energy E_b_ calculated for CX[4]-N_2(endo)_ is stronger than of CX[4]-N_2(exo)_. Also, we note that the more stable complex is characterized by a binding energy value equal to 18.90 kcal/mol. However, we show a single O⋯H stretching band position in the infrared spectrum of the CX[4]-N_2_ (N_2_ in parallel position). For CX[4]-NO_2_, we show that the NO_2_ is placed in the area of the O–H link network of the phenolic groups in the two CX[4]-NO_2(exo)_ complexes. The CX[4]-NO_2(endo)_ is specified by the energy binding equal to 20.54 kcal/mol. The CX[4]-NO_2(endo)_ has the highest E_b_ value in comparison to CX[4]-CO_2(exo/endo)_. This stability may be explained by the lowest dipole moment. The IR spectrum shows that this complex has a single O–H stretching band. The same results are obtained from CX[4]-N_2(exo)_ and CX[4]-CO_2(endo)_. Finally, we demonstrate that the stabilization of these complexes is explained by the formation of the dipole-dipole interactions between the host and the guest.

We have used the NO_3_, NO_2_, CO_2_ and N_2_ gas to be encapsulated by the CX[4] molecules [26, 27, 28, 29, 30]. We have carried out several gas capture tests inside and outside the cavity. Moreover, we conclude that the host-guests complexes given in Figure 2 are the most stable. The specific gas studied in the endo-vs. exo-cavity region will be very important to explain several electronic properties. Moreover, it is useful for the chemist to understand the photo-physical proprieties of these new endo-vs. exo-CX[4] complexation. There is a very recent work that has used a TFSI^−^ molecule as a guest for the β-cyclodextrin molecule [21]. In the same context, we try to test the interaction of these gases with the CX[4]. We note that these complexes can be a solution for new applications in the future.

The local and global reactivity parameters have been stimulated by the Fukui function theory [32], such as these parameters can be calculated by two methods; Frontier molecular orbital (FMO) or charge distribution for q = +1, q = 0 and q = -1 using the equations of Fukui:

(2) $$f_{A}^{+}=q\left(N+1\right)-q\left(N\right)$$

(3) $$f_{A}^{-}=q\left(N\right)-q\left(N-1\right)$$

(4) $$f_{A}^{0}=\frac{1}{2}\left(q\left(N+1\right)-q\left(N-1\right)\right)$$

In this context, first, we attempt to find out the reactive sites for electrophilic and nucleophilic attacks of CX[4]-gas by means of Fukui function. The approximate form of Fukui function based on atomic charge distribution will be used here. Commonly, there is no evident qualitative difference between the dual descriptor evaluated based on charge electron density of the three states (N+1, N, N-1) and the one based on the charge spin density of the two states (N+1, N-1). We have calculated the electronic parameters Softness (S^0^, S^−^ and S^+^), hardness (h^0^, h^−^ and h^+^) by using the Fukui functions (f^0^, f^−^ and f^+^) by the orbital aspect. In addition, we have investigated the QTAIM topological parameter of the endo-vs. exo-CX[4]-gas complexes to understand the nature of the interactions of these supra-molecular complexes in detail and the nature of bonds, particularly the cooperativity of hydrogen bonding of each system.

**Figure 3:**
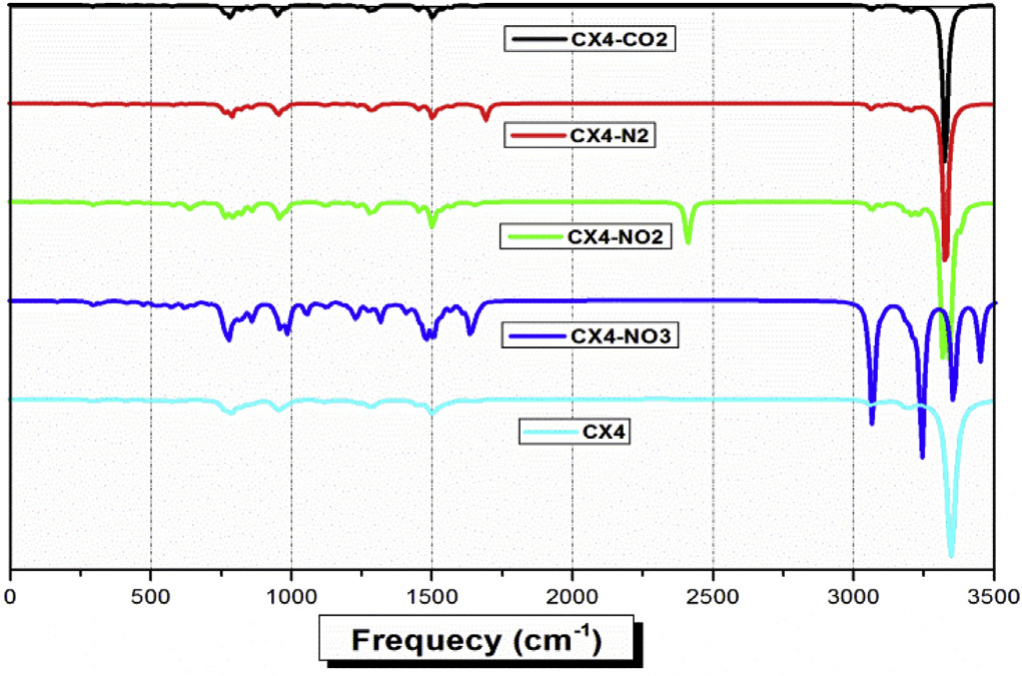
Infrared spectra of the stable CX[4]-gas complexes.

The study of the infrared spectrum and the linear polarizability (α_0_) [5, 33] explained the frequency shifting phenomena for CX[4]-CO_2_, CX[4]-N_2_, CX[4]-NO_2_ and CX[4]-NO_3_ complexes (Figure 3 and Table 2). These organic compounds can be used in optical switching, optical logic and optical interconnections for developing new technologies. The harmonic frequencies have been calculated for different complexes in order to show the effect of the encapsulation of host gases in the CX[4] cavities. The red-shifted of the O–H stretching vibrations found in (CX[4]-NO_3,_ CX[4]-NO_2,_ CX[4]-CO_2_, and CX[4]-N_2_) are compared to the free CX [4] one. In Figure 3, we have plotted the infrared spectra of the four stable host-guest complexes. In the literature, Furel et al. [35, 36] have been studied the experimental infrared spectrum of the free CX[4] molecule. The experimental spectrum shows O–H stretching vibration in the region varying from 2900 to 3500 cm^−1^. This region is characterized by five stretching vibrational bands around 3254 (υ_O-H_), 3168 (υ_O-H_), 2951 (υ_CH2_) and 2916 cm^−1^ (υ_CH2_ sym), respectively.

**Table 2:** Polarizability (α_0_) and first order hyperpolarizability (β_0_) values of the CX[4]-gas complexes calculated at B3LYP-D3BJ/6-31+G(d) level of theory.

CX[4]-gas	CX[4]-CO_2_	CX[4]-N_2_	CX[4]-NO_2_	CX[4]-NO_3_
α_0_ (10^−24^ esu)	50.12	52.55	56.41	58.28
β_0_ (10^−33^ esu)	2521.41	2543.54	2574.83	2586.62

Polarizability (α_0_): 1 a.u. = 0.1482 × 10^−24^ esu, for hyperpolarizability (β_0_): 1 a.u. = 8.6393 × 10^−33^ esu.

To take into account the an-harmonic effect, we have scaled our calculated frequencies by 0.956. The region between 3250 and 3400 cm^−1^ is characterized by the O–H stretching vibration. However, the CX [4]-N_2_ complex have two peaks around 3177 cm^−1^ and 3181 cm^−1^, respectively. These bands may be assigned to the O–H asymmetric vibrations. The same result for the CX[4]-CO_2_ complex. Furthermore, the band located at 3160 cm^−1^ is due to the degenerate of the H-bonding vibration of the phenolic O–H groups. The H-bonding between the phenol groups in the CX[4]-CO_2(endo)_ and CX[4]-N_2(exo)_ complexes are affected by the incorporation of CO_2_, and N_2_ gases. This fact is explained by the red-shift of the O–H stretching band in each compound. Contrarily, the CX[4]-NO_2_ complex has a peak located at 3170 cm^−1^ corresponding to the vibration of the O–H asymmetric band. One may observe another peak in the region of 3193 cm^−1^ (O–H asym. stretching vibration). In addition, the CX[4]-NO_2_ complex is characterized by several C–H stretch bands located less than 1800 cm^−1^. From Figure 3, we show that the interactions of the NO_3_ gas with the CX[4] lead to a split of the O–H peak to four peaks. These later are located in the vicinity of 2928, 3100, 3204 and 3298 cm^−1^, respectively. Also, we show that the CX[4]-NO_3_ complex has a very red-shifted O–H band in comparison to the others complexes. Finally, we note that the region between 600 and 1800 cm^−1^ is specified by the C_ar_-H, C_meth_.-H and C=C stretching vibrations in the CX[4]-NO_2_ and CX[4]-CO_2_ complexes.

Likewise, we demonstrated that a region appears in the CX[4]-NO_3_ complex less than 1800 cm^−1^ is characterized by several peaks corresponding to the C_arom._-H and C_meth._-H stretching vibrations. We have noted that the red-shifted values between the CX[4]-gas complexes are around to 44 (CX[4]-NO_3_), 24 (CX[4]-NO_2_), and 9 cm^−1^ (CX[4]-CO_2_), respectively. In conclusion, the red-shift of the O–H stretching bands explain the sensibility of the gas to encapsulate in the inside cavity of the CX[4] molecule. From Table 2, we may deduce that the polarizability values of the CX[4]-CO_2_, CX[4]-N_2_ and CX[4]-NO_2_ are approximately between 50 × 10^−24^ and 58 × 10^−24^ esu. In addition, these values are almost 10 times higher than the value of the prototype molecule (The α_0_ of urea is equal to 5 × 10^−24^ esu).

Also, it is very clear that the CX[4]-NO_3_ complex may be a good candidate of nonlinear optical applications, this complex is characterized by a polarizability almost to 60 × 10^−24^ esu. The value of the polarizability (α_0_) of the CX[4]-NO_3_ explained the red-shifting of the H-binding stretching vibration. The greater polarizability of the three host-guests relative to the urea may be explained by the charge transfer between the gases to the π-electron of the phenol ring.

**Figure 4:**
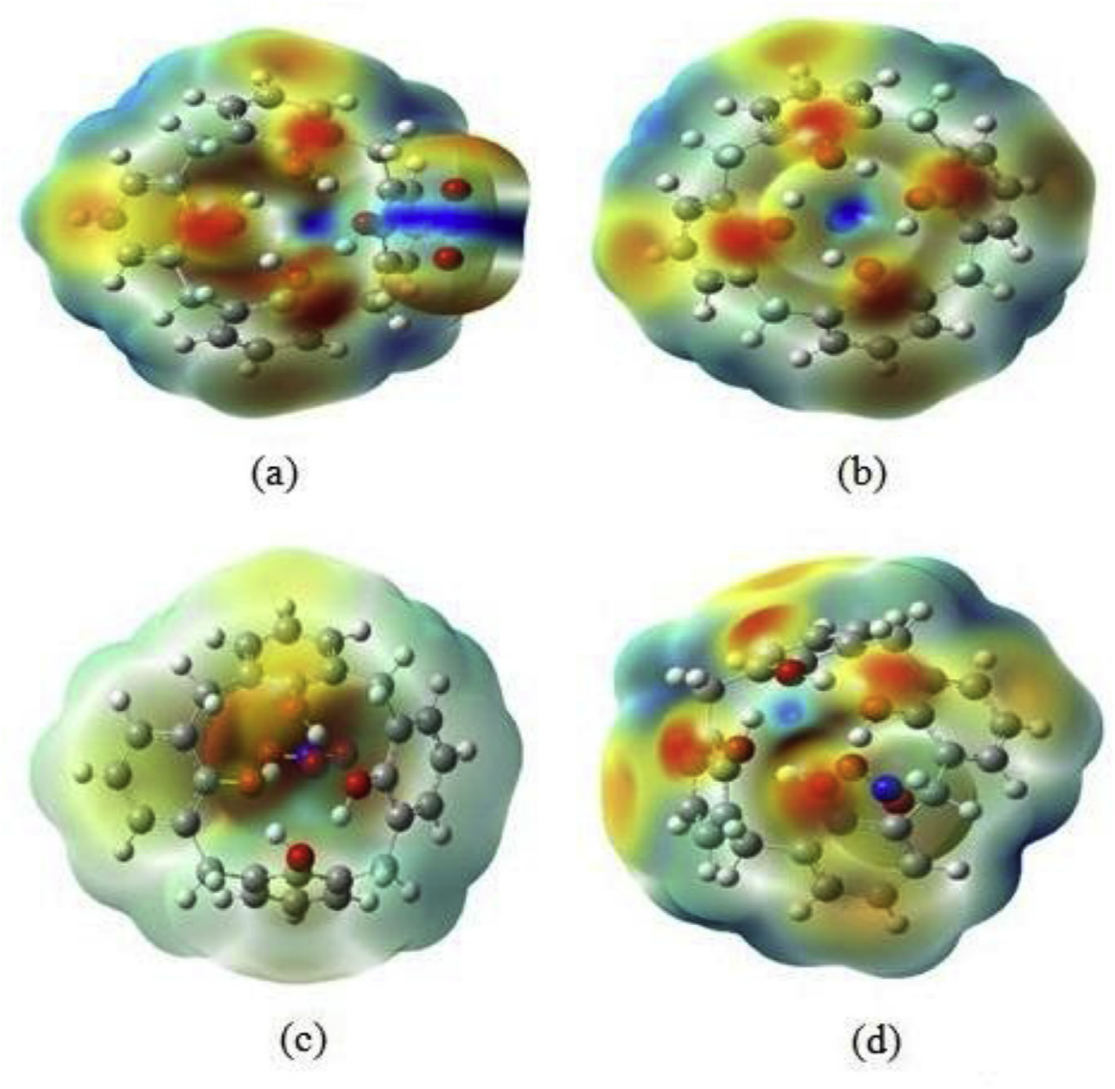
MEP analysis of the stable CX[4]-gas (CX[4]-CO_2_ (a), CX[4]-N2 (b), CX[4]-NO_2_ (c) and CX[4]-NO_3_ (d)) complexes calculated by B3LYP-D3BJ/6-31+G(d) level.

Understanding the relative affinity of each stable host-guest complexes is very important to known the specific properties of atoms. For this idea, we have calculated the local and global softness or hardness, recently proposed by Franco-Pérez et al. [37]. These reactive parameters have been used to take into account the distribution of electrophilic and nucleophilic active sites. All these reactive parameters are calculated in the same framework. The studied systems have been chosen to test the new index of the Softness/hardness parameters in these specific regions. Indeed, regarding the MEP graphs (Figure 4), it is the zone with V(r) < 0 that we are interested in. For both CX[4]-gas interactions, with V(r) > 0 where an electrophilic zone and V(r) < 0 is a nucleophilic zone. The results are illustrated in Table 3. To better understand the sites to interpret (See Figure 2 and Table 3). For the explanation of the reactive interactions of the CX[4]-gas complexes using the atomic charge distribution, we have taken into account the Fukui function in each site. From Table 3, we can deduce that, the O_1_ atom has the strongest nucleophilic sites in the CX[4]-CO_2_ complex with the highest S^−^ connect to the H_1._ However, S^−^ values for H_2_ is the biggest than the S^−^ of H_1_.

**Table 3:** Condensed to atom: Fukui function, local softness, local hardness and relatives softness and relatives hardness values of reactive atom of CX[4]-gas complexes.

		f^-^	f^+^	f^0^	S^-^	S^+^	S^−^/S^+^	h^-^	h^+^	h^−^/h^+^
CX [4]-CO_2_	O1	0.0308	-0.0028	0.0139	0.0039	-0.0004	-9.7500	0.4941	-0.0287	-17.2160
	O2	0.00001	-0.0001	-0.00008	0.000002	-0.00002	-0.1000	0.0015	-0.0014	-1.1232
	O3	0.00004	-0.00008	-0.00001	0.000006	-0.00001	-0.6000	0.0014	-0.0007	-1.9985
	H1	-0.00005	0.0014	0.00069	-0.000008	0.00019	-0.0421	-0.0118	0.0115	-1.0260
	H2	0.00005	0.0026	0.00135	0.000007	0.00034	0.0205	-0.0192	0.0211	-0.9099
	C1	-0.0002	0.0014	0.00059	-0.000025	0.00018	-0.1389	-0.0133	0.0110	-1.2090
CX [4]-N_2_	O1	0.0455	-0.0029	0.0213	0.0059	-0.00038	-15.5261	0.7194	-0.0333	-21.6036
	H1	0.0003	0.0013	0.00078	0.000029	0.00017	0.1705	-0.0068	0.0106	-0.6409
	N1	0.0000	0.000005	0.000002	0.00000	0.000001	0.0000	-0.000003	0.00003	-0.1000
	N2	0.000004	0.000007	0.000005	0.000001	0.000001	1.0000	0.000013	0.00005	0.2600
CX [4]-NO_2_	O1	0.000001	0.2096	0.1305	0.00000	0.0392	0.0000	-1.9727	1.5044	-1.3111
	O2	-0.00003	0.2605	0.1302	-0.000004	0.0391	-0.000102	-1.9696	1.5017	-1.3116
	O3	0.0551	0.00009	0.0277	0.0083	0.000015	553.3333	0.7817	0.0499	15.6504
	H1	0.0004	-0.00004	0.00015	0.000052	-0.000006	-8.6666	0.0054	0.00007	77.0000
	N1	0.00006	0.4674	0.2337	0.000008	0.0702	0.00011	-3.5322	2.6943	-1.31103
CX [4]-NO_3_	O1	-0.00007	0.0004	0.00017	-0.000009	0.000053	-0.1698	-0.0039	0.0029	-1.33525
	O2	0.0003	-0.0028	-0.0013	0.000032	-0.00037	-0.0864	0.0244	-0.0206	-1.1850
	O3	-0.0006	-0.0054	-0.0029	-0.000069	-0.00072	0.0958	0.0326	-0.0401	-0.8121
	O4	0.1103	-0.0004	0.0549	0.0147	-0.000052	-282.6923	1.6572	-0.0011	-1506.6
	N1	-0.0002	0.0010	0.0042	-0.000026	0.00014	-0.1857	-0.0106	0.0077	-1.3903
	H1	0.0016	0.00017	0.00089	0.00022	0.000022	10.0000	0.0231	0.0013	18.4880

This zone is characterized by the strongest attraction of electrons (electrophilic region). The S^−^/S^+^ values of the specific atoms are more descriptive for this gas. Concerning the orbital interaction in the CX[4]-N_2_ gas, the oxygen atom O_1_ has the strongest nucleophlie with the least value S^−^/S^+^ ratio in comparison with N_2_ atoms (see Table 3). As expected, this atom seems decreasing the nucleophilicity to the center of the cavity.

In addition, the O_3_ atom includes the maximum nucleophilic sites with the highest value of S-/S+ ratio in comparison with other specific atoms (see Figure 2 and Table 3). For the CX[4]-NO_3_ complex, we find that the O_3_ atom exhibits a large nucleophilic site characterized by the highest values of S^−^/S^+^. The minimum of the repulsive electron has been surrounded by the O_4_ atom. This information explained that the maximum of nucleophilic region co-exists in the center of the cavity. The NO_2_ gas is the hardest guest with a global/local hardness value equal to 0.7871 and 0.0499 respectively. This guest is characterized by the h^−^/h^+^ values equal to 15.6504. The nucleophilic minimum sites exist in the CX[4]-NO_3_ complex, such as the O_4_ atom that has a h^−^/h^+^ ratio values equal to -1506.609. Looking again Table 3, O_3_ in the center of the cavity of the CX[4]-NO_2_ complex explains well the strong electrostatic interaction of NO_2_ gas with the CX[4] molecule. In the CX[4]-NO_3_ complex, the most electrophilic zone (H_1_) is described by the strongest H-binding interaction. The CX[4]-NO_3_ complex exhibits the most dramatic change (RSD = 85.3%). It means that the local hyper-softness (LHS) should be a much better candidate for the correlations with the experimental catalytic activity. The explanations of the relative nucleophilicity (S^−^/S^+^ ratio), electrophilic and nucleophilic hardness/softness check that, the CX[4]-NO_2_ and CX[4]-NO_3_ complexes are very iso-electronic in comparison with the other host-guests.

**Figure 5:**
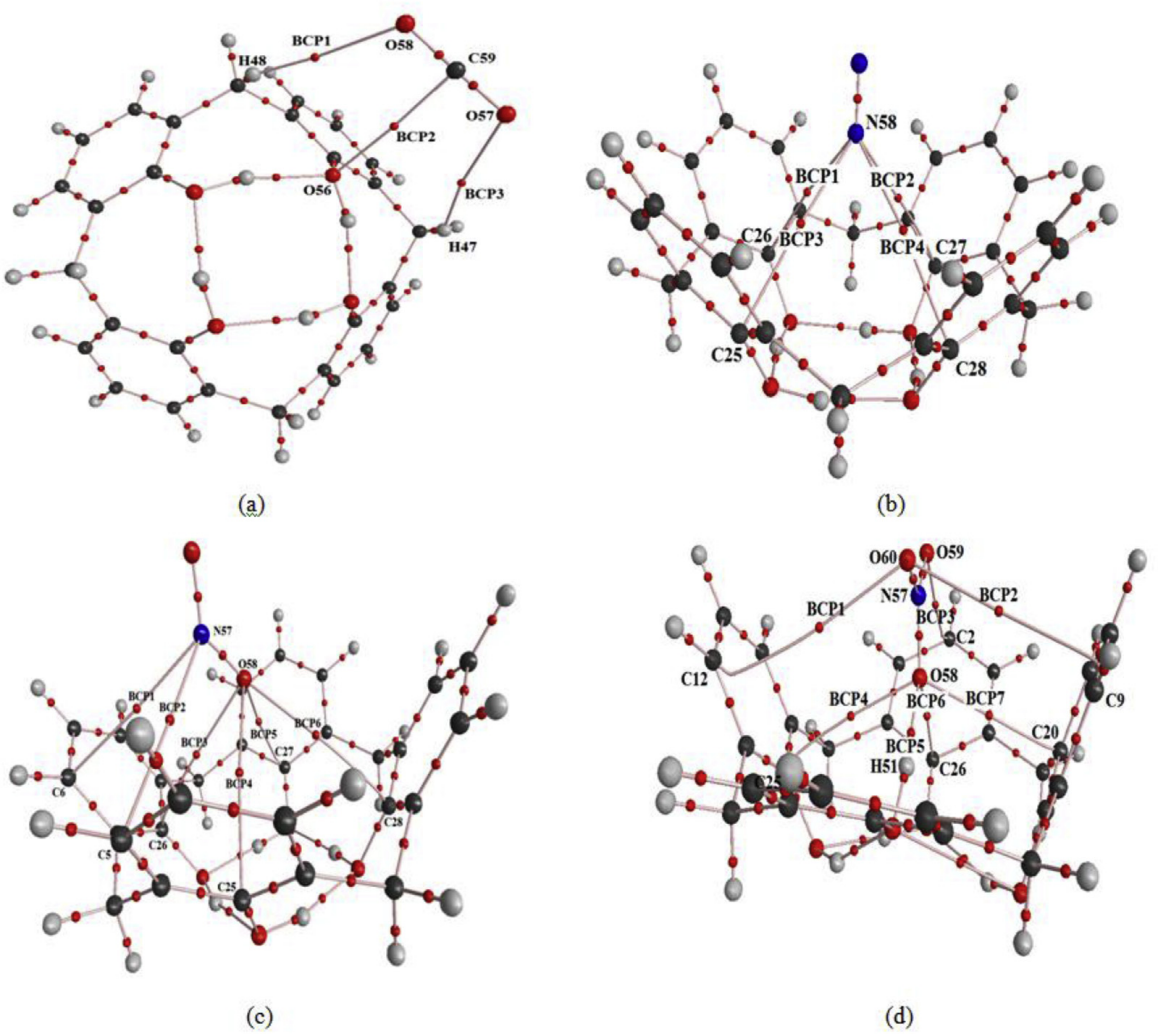
AIM molecular graphs showing the different BCPs in CX[4]-CO_2_ (a), CX[4]-N_2_ (b), CX[4]-NO_2_(c) and CX[4]-NO_3_ (d) calculated at B3LYP-D3BJ/6-31+G(d) level of theory.

The nature and the strength of the interactions between CX[4] and the specific gases have been determined by the AIM and NCI-RDG analysis. For all Bond Critical Points (BCPs) caused by the encapsulation of the host gases in CX[4], we have calculated the chief topological parameters; the electron density (ρ) and it's laplacian (∇^2^ρ) by the Atom In Molecule (AIM) theory. Other topological parameters are also extracted, such as the kinetic energy density (G), the Hamiltonian kinetic energy (H), the interaction energy (E_X...Y_) and the ellipticity (ε). The AIM molecular graphs have been shown in Figure 5 the different BCPs in the CX[4]-gas complexes.

The topological parameters calculated at selected BCPs are listed in the Table 4. One may see that for the CX[4]-CO_2_, the electron density values are found between 0.0021 to 0.0064 a. u. and the Laplacian is negative. The BCP_2_ of this complex has a highest electron density value. The interaction energy (E_BCP2_) is equal to -5.614 kJ/mol. Also, we note that the ellipticity is equal to1.67 a. u. which indicates that the C_59_⋯O_56_ is an instable interaction. Moreover, the CX[4]-N_2_ complex is characterized by the existence of four BCPs. The electron density values in these BCPs of this complex are around to 0.0020 a. u. The Laplacian values are positive. We show that the interactions between the CX[4] and the N_2_ gas are weak, which is proved by the N58⋯O26 and N58⋯O27 interactions. The H-bonding energy in these BCPs is equal to -1.296 kJ/mol. We have noted that the CX[4]-NO_2_ complex is characterized by a weak intermolecular interactions.

**Table 4:** Topological parameters: electron density ρ(r), Laplacian of electron density ∇^2^ρ(r), electronic kinetic energy density G(r), total electron energy density H(r), the eigenvalues λ_1,_ λ_2,_ λ_3_, ellipticity of electron density ε and interaction energy E_X…Y_(r) in kJ/mol.

	BCPs	ρ (a.u)	Δ^2^ρ (a.u)	G (a.u)	H (a.u)	E_X..Y_ (KJ/mol)	λ_1_ (a.u)	λ_2_ (a.u)	λ_3_ (a.u)	⎜λ_1_⎜/λ_3_	ε
CX [4]-CO_2_	BCP1	0.0029	-0.0029	0.0022	0.0007	-1.968	-0.0022	-0.0021	0.0160	0.1375	0.05
	BCP2	0.0064	-0.0070	0.0057	0.0014	-5.614	-0.0056	-0.0021	0.0358	0.1564	1.67
	BCP3	0.0021	-0.0022	0.0016	0.0006	-1.337	-0.0016	-0.0011	0.0115	0.1391	0.45
CX [4]-N_2_	BCP1	0.0021	-0.0017	0.0014	0.0004	-1.296	-0.0012	-0.0004	0.0086	0.1395	2.00
	BCP2	0.0020	-0.0017	0.0013	0.0004	-1.296	-0.0012	-0.0004	0.0085	0.1412	2.00
	BCP3	0.0021	-0.0017	0.0013	0.0004	-1.288	-0.0012	-0.0004	0.0085	0.1412	2.00
	BCP4	0.0020	-0.0017	0.0013	0.0004	-1.271	-0.0012	-0.0004	0.0084	0.1429	2.00
CX [4]-NO_2_	BCP1	0.0025	-0.0019	0.0016	0.0004	-1.576	-0.0014	-0.0007	0.0097	0.1443	1.00
	BCP2	0.0024	-0.0018	0.0015	0.0003	-1.500	-0.0013	-0.0006	0.0093	0.1398	1.17
	BCP3	0.0026	-0.0023	0.0019	0.0005	-1.816	-0.0017	-0.0007	0.0117	0.1453	1.43
	BCP4	0.0026	-0.0023	0.0018	0.0005	-1.775	-0.0017	-0.0007	0.0115	0.1478	1.43
	BCP5	0.0019	-0.0019	0.0015	0.0004	-1.386	-0.0012	-0.0004	0.0093	0.1290	2.00
	BCP6	0.0019	-0.0019	0.0014	0.0004	-1.319	-0.0011	-0.0004	0.0090	0.1222	1.75
CX [4]-NO_3_	BCP1	0.0016	-0.0015	0.0012	0.0004	-1.019	-0.0007	-0.0002	0.0071	0.0986	2.50
	BCP2	0.0023	-0.0021	0.0016	0.0004	-1.538	-0.0012	-0.0003	0.0097	0.1237	3.00
	BCP3	0.0064	-0.0048	0.0040	0.0008	-4.280	-0.0047	-0.0018	0.0256	0.1836	1.61
	BCP4	0.0072	-0.0057	0.0050	0.0007	-5.578	-0.0054	-0.0018	0.0300	0.1800	2.00
	BCP5	0.0375	-0.0302	0.0309	-0.0006	-41.348	-0.0612	-0.0579	0.2400	0.2550	0.06
	BCP6	0.0194	-0.0154	0.0141	0.0014	-16.671	-0.0175	-0.0128	0.0920	0.1902	0.37
	BCP7	0.0079	-0.0063	0.0056	0.0007	-6.428	-0.0053	-0.0029	0.0334	0.1587	0.83

**Figure 6:**
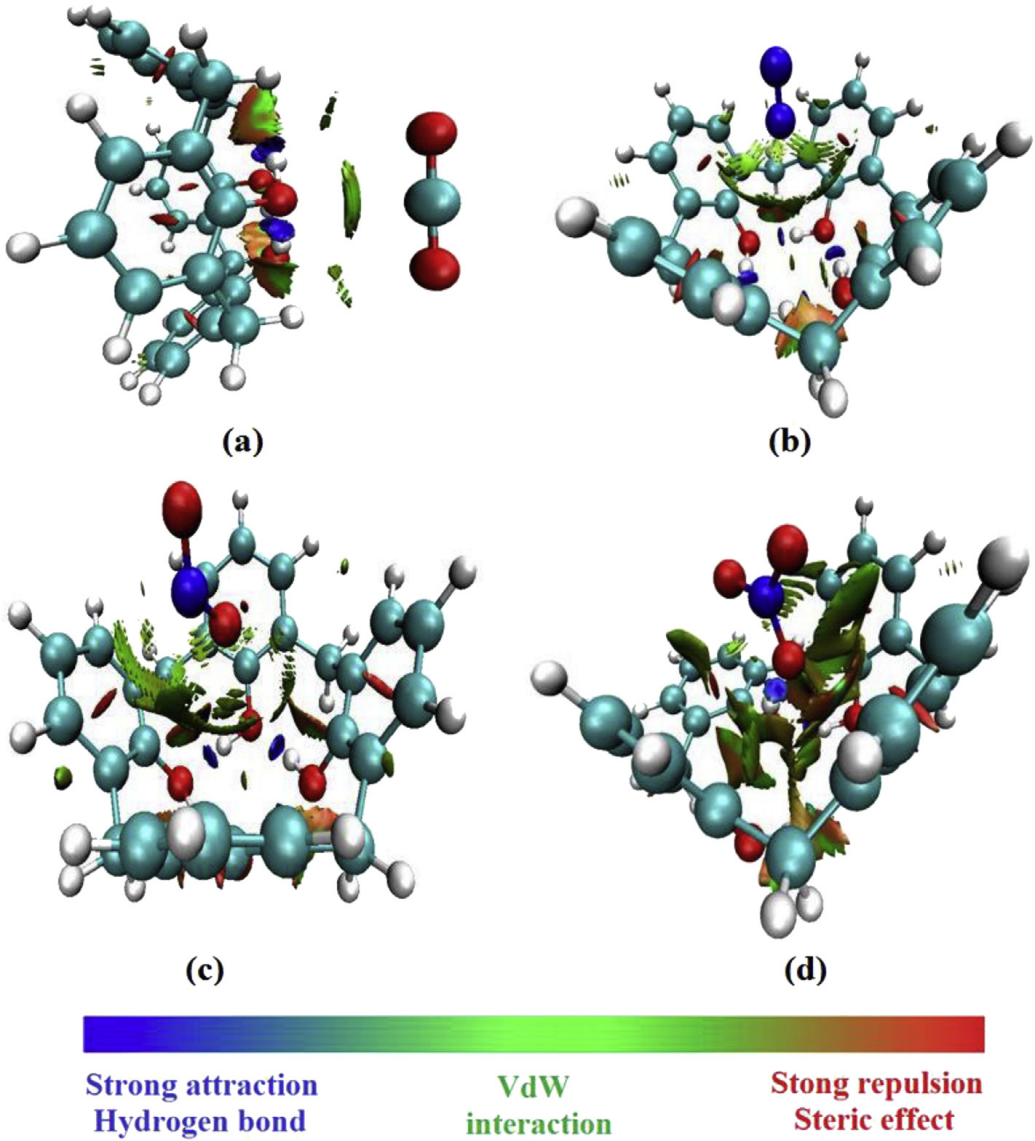
NCI-RDG plots of the electron density and its reduced gradient of the inclusion complexes for CX[4]-gas (CX[4]-CO_2_(a), CX[4]-N_2_(b), CX[4]-NO_2_(c) and CX[4]-NO_3_(d)). The iso-surfaces were constructed with RGD = 0.5 a. u and the colors scaling from -0.01 to -0.01 a. u.

The value of the interaction energy in the BCP_3_ is worth to1.82 kJ/mol. In this BCP, we can deduce the height stability of the interaction between O_58_ and C_26_ atoms by the low value of the ellipticity ε = 1.43. The AIM topological parameters for the CX[4]-NO_3_ complex demonstrated that this complex has the highest interactions at the level of the BCP_5_ and BCP_6_. Moreover, the electron density values in these interactions are equal to 0.0375 and 0.0194 a. u., respectively. The ellipticity value is equal to 3 in these BCPs. This result has explained that the interaction between O_60_ and C_9_ is less stable in comparison with other inter-atomic interactions. On the other side, we notice that the H-bonding interaction O_58_⋯H_51_ has a weak value of ellipticity equal to 0.06. In addition, this result confirm that this H-bonding interaction is very stable than others. This idea proves that the interaction between the host and the guest is related to the stability of the inter-atomic interactions. The NCI-RDG analysis shows that the CX[4]-NO_3_ complex is characterized by the existence of weak Van der Waals interaction (green color) between O_60_⋯C_12_, O_60_⋯C_9_, O_58_⋯C_20_ and O_58_⋯C_25_ (Figure 6).

We found that this complex shows the existence of the H-bonding type interaction between the O_58_ atom and H_51_ atom (blue color). We have noted that the CX[4]-NO_3_ complex shows that the O_58_⋯C_26_ interaction (NCI graphs) reflect the larger stability of this host-guest. This result is well confirmed by the AIM theory. The AIM and the NCI-RDG analyses have demonstrated that the presence of attractive and repulsive interactions between the gas and the CX[4] molecule is very necessary for the stability of the encapsulated complexes inside of its cavity.

## 4 Conclusion

The encapsulation of NO_3_, NO_2_, CO_2_ and N_2_ gases in the CX[4] cavities have been investigated by using DFT calculations. The optimization of the studied complexes has shown that the position of the gas inside the cavity is very stable than that outside of the cavity. This fact is clearly explained by the distribution of electrophilic and nucleophilic active sites. The infrared spectrum and the polarizability study have explained the role of the NO_3_ gas in the red shifted of the O–H band in comparison with the other gases. The local softness and hardness parameters of the various inclusion complexes specified the high rigidity and conductivity of CX[4]-NO_3_ in comparison with the other complexes. The AIM analysis has shown clearly the strong interactions of the gas NO_3_ and NO_2_ with the endo-cavity environment of the CX[4].

## Declarations

### Author contribution statement

B. Gassoumi, H. Ghalla, R. Ben. Chaabane: Conceived and designed the analysis; Analyzed and interpreted the data; Contributed analysis tools or data; Wrote the paper.

### Funding statement

This research did not receive any specific grant from funding agencies in the public, commercial, or not-for-profit sectors.

### Competing interest statement

The authors declare no conflict of interest.

### Additional information

No additional information is available for this paper.
